# Editorial: Advancing muscle health: from technical and clinical research to practice

**DOI:** 10.3389/fresc.2026.1885911

**Published:** 2026-06-11

**Authors:** Katie L. Boncella, Micah Brown, Jonathan P. Beausejour, Michael O. Harris-Love

**Affiliations:** 1 Muscle Morphology, Mechanics, and Performance Laboratory, School of Medicine, University of Colorado Anschutz Medical Campus, Aurora, CO, United States; 2 Department of Physical Medicine and Rehabilitation, University of Colorado Anschutz Medical Campus, Aurora, CO, United States; 3 Department of Biomedical Engineering, University of Colorado Denver Anschutz Medical Campus, Aurora, CO, United States; 4 Physical Therapy Program, School of Medicine, University of Colorado, Aurora, CO, United States; 5 Eastern Colorado VA Geriatric Research, Education, and Clinical Center, Aurora, CO, United States; 6 Research Service, VA Eastern Colorado Healthcare System, Aurora, CO, United States

**Keywords:** consensus, implementation, morphology, muscle health, muscle performance, strength, tissue composition

## 1 Introduction

The manuscripts that comprise the Research Topic issue, *Advancing Muscle Health: From Technical and Clinical Research to Practice*, highlight “muscle health” as a multidimensional construct rather than a single outcome measure (1). Skeletal muscle is a multifunctional organ involved in force production, metabolic regulation, endocrine signaling, postural control, and immune modulation (2, 3). Declines in muscle health contribute to sarcopenia, frailty, falls, metabolic dysfunction, and loss of independence, imposing substantial personal and societal burdens. Recent literature reveals substantial conceptual overlap in core domains of muscle health, yet wide variation in recommended tools, measurement cutoff or threshold values, and the interpretation of outcome measures (4). This heterogeneity limits reproducibility, comparability across studies, and routine clinical adoption.

Various groups have addressed the need for a common typology and framework for muscle health (4–6). Boncella et al. (2025) synthesize this landscape into a practical conceptual framework grounded in the World Health Organization's International Classification of Functioning, Disability and Health (ICF) (7). By linking body systems/structures to individual participation, the framework provides rehabilitation professionals (e.g., physical therapists, physiatrists, occupational therapists) with a language and structure that directly informs activities of daily living (ADLs) and functional independence. The collective research within this field strengthens this call to action. The purpose of this editorial is to move beyond individual models through synthesizing their alignments and distinctions, then translating the integrated conceptual foundation into practical implementation steps for rehabilitation practice. This implementation roadmap emphasizes feasibility, standardization, and measurable impact on functioning.

## 2 A rehabilitation perspective on the muscle health framework

Terms that characterize skeletal muscle, such as muscle health or muscle quality, are inherently pluralistic constructs that cannot be reduced to any single physiological and/or clinical framework. Skeletal muscle exerts wide- system effects, and its operational definition varies depending on the domain of interest (e.g., force production, metabolism, thermoregulation, or myokine signaling) (2, 8). No single endpoint measure serves all domains equally well, and the domain(s) of interest logically determines which questions are viable, which tools are appropriate, and what constitutes a meaningful outcome. This domain-based perspective untethers muscle health assessment from lean body mass–driven approaches alone and clarifies the distinction between measurement methods and targeted physiologic domains.

Building on this foundation, Boncella and colleagues proposed a framework for muscle health that was derived from accepted definitions of physical health and operationalized using components from the ICF model. The ICF has been broadly adopted by physical rehabilitation practitioners and investigators. Consequently, this approach does not directly address myokine signaling, thermoregulation, or metabolic homeostasis as primary endpoints. Rather, it proposes formal components to guide the selection of outcomes that are well-suited for rehabilitation while also incorporating outcomes with utility across domains:

- Body and Tissue Composition (mass, architecture, fatty infiltration, fibrosis)
- Muscle Performance (strength, power, rate of force development)
- Functional Task Performance (gait, balance, transfers, endurance)

This multidimensional approach provides a more complete picture than any singular outcome measure. Outcomes associated with these components map onto the ICF model inclusive of body systems/structures, activities, and participation, enabling clinicians to proactively and systematically assess muscle health in the context of patient-centered care. This ICF orientation is particularly advantageous for rehabilitation professionals, as it prioritizes how muscle changes translate into mobility, balance, falls risk, and participation restrictions, while still allowing for integration of clinically relevant environmental and personal factors. However, it should be noted that domain pluralism (the acknowledgement that distinct scientific disciplines and health professions approach muscle health through different theoretical lenses) is not without its challenges. The boundaries between physiological domains are heuristically useful but biologically fraught: myokine signaling shapes glucose metabolism, which modulates force production capacity, which in turn governs activity levels and myokine expression.

These interdependencies suggest that the domains are not fully separable, but rather constitutive of one another. Moreover, while disciplinary pluralism has very practical advantages for researchers, it risks posing operational barriers in clinical and public health contexts where shared constructs, common staging criteria, and cross-disciplinary communication are practical necessities. Subsequently, our collective task includes identifying frameworks that incorporate outcome measures that retain meaning across domains without collapsing their distinctions. While the three components of body and tissue composition, muscle performance, and functional task performance are considered together within the muscle health framework, the individual outcome measures associated with each component (e.g., imaging indices, force production metrics, and functional performance tests) remain available as modular tools that other disciplines may adopt within their own theoretical frameworks and apply toward different interpretive purposes.

Nevertheless, persistent barriers remain even when differing frameworks align: lack of universal cut-offs for muscle composition estimates, force/power normalization approaches, method-dependent estimates of muscle mass, and underreporting of protocol details (e.g., device settings, patient positioning, and operator experience. Table 1 conveys these challenges by cataloguing modalities, technical/protocol factors, primary measures, illustrative cut-offs, and interpretation nuances across commonly used measurement tools. Even as conceptual frameworks for muscle health continue to evolve, a persistent barrier remains: not the absence of measures, but the lack of a shared structure within which those measures can be meaningfully compared and interpreted.

**Table 1:** Measurement approaches and methodological considerations across muscle health domains.

Category	Modality/Method	Technical and Protocol Factors	Primary Measures	Typical Cut-offs (selected major criteria)^a^	Interpretation (Quantity/Quality)
Body or tissue composition	DXA	Manufacturer/model x-ray beam geometries, scan mode, software version, ROI selection, patient positioning	ALM, ALM/height^2^, SMI, total FFM	EWGSOP2: ALM/height^2^ < 7.0 kg/m^2^ (♂), <5.5 kg/m^2^ (♀) (9)	Primarily quantity; low values indicate low muscle mass/ sarcopenia risk.
	BIA	Device manufacturer/model, frequency type (single- vs. multi-frequency), electrode configuration, patient positioning, hydration status, time of day, skin/electrode contact	FFM, SMI, skeletal muscle mass, ALM, phase angle	Varies by device/equation; phase angle <4.5–5.0° often indicates poorer health (10)	Estimates quantity; lower SMI+lower phase angle → poorer muscle quantity and quality.
	MRI	Manufacturer/model, field strength (1.5 T vs. 3 T), sequence type (T1/T2/Dixon), voxel size/ resolution, slice thickness/gap, TE/TR parameters, coil placement, patient positioning/ breath-hold, post-processing algorithms	Muscle CSA/ volume, fat fraction (%), intramuscular fat, T2 relaxation time (ms)	No universal cut-offs; fat fraction >10%–15% often considered elevated	Excellent for quantity (volume/CSA) and quality (fatty infiltration, T2 reflecting edema/fibrosis).
	Ultrasound	Probe frequency (MHz) and type, probe placement/pressure, gain settings, depth/range, device-specific TGC, angle of incidence/ tilt, patient positioning, acute post-exercise muscle swelling/edema anatomic site/ROI consistency, operator experience	Muscle thickness, CSA, EI, fascicle length, pennation angle	No universal cut-offs; EI > upper tertile or device-specific reference (11)	Quantity (thickness/CSA) and quality (higher EI = more fat/fibrosis).
	CT	Scanner model, tube voltage (kVp), tube current (mAs), slice thickness (partial voluming), reconstruction kernel, IV contrast phase/timing, FOV/matrix, patient positioning, breath-hold, ROI/segmentation/ thresholds, software variations	CSA, SMI, muscle attenuation (HU), IMAT area/volume	SMI: <42–52 cm^2^/m^2^ (♂), <33–38 cm^2^/m^2^ (♀) varies by criteria; HU <30–40 indicates myosteatosis (12, 13)	Quantity (CSA/SMI) and quality (lower HU/higher IMAT=poorer quality/ myosteatosis).
Muscle performance	Hand Grip Strength	Dynamometer type/model, handle position, body posture, arm/elbow/ wrist position, hand tested (dominant/non-dominant), number of trials, rest intervals, verbal encouragement, effort duration, recent activity/fatigue	Absolute (kg), relative to body mass (kg/kg) or BMI (kg/kg/m^2^)	EWGSOP2: <27 kg (♂), <16 kg (♀); (9) FNIH: <26–30 kg range depending on criteria (14)	Global strength proxy; low values indicate reduced overall neuromuscular function/sarcopenia risk.
	Knee Extension Strength	Mode of testing, muscle action tested, device manufacturer, knee angle, hip angle, stabilization method, number of trials, max vs. averaged data, rest intervals, verbal encouragement, effort duration, limb tested, recent activity/warm-up	Peak torque (Nm or kg), relative to body mass or muscle mass	Limited universal cut-offs; often <1.0–1.7 Nm/kg body mass or device-specific (15, 16)	Key lower-limb strength measure; strongly linked to function (gait, chair rise); normalized torque reflects quality.
Functional task (22)	Gait Speed	Pace (habitual vs. fast), distance (4 m vs. 6 m), starting protocol (static vs. dynamic start) and acceleration zones, timing method (stopwatch vs. automatic), assistive device use, surface, number of trials, verbal instructions/ encouragement, recent activity	Time (s), velocity (m/s), use of stature as unit of measure	EWGSOP2: ≤0.8 m/s (slow); ≤1.0 m/s often used	Reflects mobility, endurance, lower-limb power; slow speed=high sarcopenia/frailty risk.
	Sit-to-Stand	Chair height, arm use and starting position, instruction/pace, reps/ timing (5 reps timed vs. reps in 30 s), verbal encouragement, number of trials, rest intervals, assistive device, recent activity/fatigue	Time for 5 rises (s), number in 30 s	5-rep time >15 s or <12–15 rises in 30 s often indicative of impairment	Lower-limb power, balance, transfer ability.
	Timed Up and Go	Chair height, armrest use, instruction/pace, starting position, verbal instructions/encouragement, practice trial and number of trials, rest intervals, assistive device, turning strategy, surface, recent activity/cognition, timing method (e.g., stop on back contact)	Time (s)	>12–20 s indicates increased fall risk/ mobility limitation	Dynamic balance, fall risk, functional mobility.
	Balance	Side-by-side, semi-tandem, full tandem; stance progression, hold duration, arm position, footwear/ surface, starting assistance, verbal instructions, demo and practice, safety spotting, number of attempts, recent activity/cognition/fatigue	Hold time (s)	<10 s in tandem often indicates poor static balance	Static postural control; shorter times linked to fall risk.

a Cut-offs are illustrative and vary by guideline (EWGSOP2, AWGS, FNIH, etc.); always consult the specific reference population, device, and protocol.

ALM, Appendicular Lean Mass; AWGS, Asian Working Group for Sarcopenia; BIA, Bio-Electrical Impedance; BMI, Body Mass Index; cm, Centimeter; CSA, Cross Sectional Area; CT, Computed Tomography; DXA, Dual-Energy x-ray Absorptiometry; EWGSOP2, European Working Group on Sarcopenia in Older People; EI, Echogenicity; FFM - Fat Free Mass; FNIH, Foundation for the National Institutes of Health; FOV, Field of View; HU, Hounsfield Units; IMAT, Intramuscular Adipose Tissue; IV, Intravenous; kg, Kilogram; kVp, Peak Kilovoltage; m, Meter; mAs, Milliampere-Seconds; ms, Millisecond; MHz, Megahertz; MRI, Magnetic Resonance Imaging; Nm, Newton-meters; reps, repetitions; ROI, Region of Interest; s, Second; SMI, Skeletal Muscle Index; T1, T1-Weighted Image; T2, T2-Weighted Image; T, Tesla; TE, Echo Time; TGC, Time-Gain Compensation; TR, Repetition Time; ♀, female, ♂, male.

## 3 Complementary frameworks: alignment, distinctions, and rehabilitation implications

Recent frameworks converge on the need for structured, multidimensional approaches to muscle health while offering complementary strengths that support feasible and appropriate clinical implementation in rehabilitation. Boncella et al. (2025) position their model as an aspect of physical health using the ICF as an organizing framework with three core, interrelated components (body and tissue composition, muscle performance, and functional task performance) that explicitly link biological impairments to activity limitations and participation restrictions (4). In contrast, Heymsfield et al. (2023) propose a hierarchical model grounded in the principle that “outcomes follow function follow form” (the OFF rule) (6). In this framework, muscle *form* (mass, shape, composition) underpins *function* (strength, power, endurance, metabolic capacity), which in turn drives clinical *outcomes* (mobility, falls risk, independence, survival). The model draws from existing sarcopenia, malnutrition, and cachexia guidelines, advocating form and function as equivalent “gateway” nodes for screening and intervention while emphasizing that functional measures typically show stronger associations with patient-relevant outcomes. These two models align strongly: both endorse multidimensional assessment over single metrics and recognize the directional hierarchy from tissue-level properties to functional performance to participation. Integration of the ICF adds specific contextual factors (personal and environmental) and a standardized language for interdisciplinary teams, while the OFF model provides a clear pathophysiological rationale for prioritizing functional outcomes in guideline development and clinical decision-making.

Perkisas et al. (2026) characterize skeletal muscle through a temporal approach that incorporates a six-stage muscle health continuum (17). This continuum reframes sarcopenia as an advanced (Stage 5) pathological state within a lifelong trajectory that includes supraphysiological (Stage 1) and subclinical stages (Stages 3–4). This continuum also introduces functional reserve and adaptability as dynamic qualifiers, enabling earlier preventive rehabilitation and longitudinal tracking, which are elements less emphasized in the static hierarchical or domain-based models. In addition, van Zanten et al. (2026) add a clinical nutrition perspective, stressing the personalization of interventions (e.g., protein quantity/timing, anabolic support) based on an individual's position within the muscle health spectrum rather than generic recommendations (5). This approach complements all prior models by addressing modifiable external drivers that influence progression along the continuum and within the form–function–outcome pathway.

Important points of differences and alignment exist among these frameworks for rehabilitation practice include:

- Scope of focus: The six-stage muscle health continuum and OFF model lean toward a medical/biological model with etiological subtypes and staging, while the ICF-informed muscle health perspective centers rehabilitation-relevant functioning and disability.
- Prevention vs. treatment: The six-stage muscle health continuum explicitly supports proactive intervention in at-risk or subclinical individuals; the OFF model helps justify why early functional training yields outsized outcome benefits, but all of the frameworks are dependent on clear outcome measures, cutoff values, and standardized interpretation to have clinical relevance.
- Aggregate measurement: All frameworks acknowledge that form (e.g., muscle mass estimates or ultrasound thickness) is foundational but insufficient alone. While real-world performance outcomes (e.g., mobility status or gait speed) provide valuable intervention targets, functional limitations cannot be explicitly attributed to muscle dysfunction without data from body or tissue composition estimates and standardized muscle performance measures.

Together, these frameworks form a complementary ecosystem: the hierarchical OFF model (6) supplies pathophysiological logic, the ICF-informed muscle health framework (4) provides physical rehabilitation structure and typology, the six-stage muscle health continuum (17) adds temporal staging and prevention, and emerging perspectives concerning personalized interventions (5) further address modifiable factors. An appropriate synthesis of these perspectives may prove to resolve some limitations to implementation by moving from isolated domain assessment to an integrated, dynamic, person-centered approach suitable for diverse rehabilitation settings. Nonetheless, advancing muscle health frameworks is necessary but not sufficient. Realizing the clinical and research utility of muscle health assessment requires resolving the persistent variability in measurement protocols and data interpretation that currently limit cross-study comparability (Table 1).

## 4 Roadmap for clinical implementation

There has been a recent call to advance the implementation of muscle health in rehabilitation settings through the refinement of assessment guidelines (4). The following provides a roadmap to help transition from conceptual frameworks into actionable practice:

Step 1: Formalize a Process to Standardize Protocols, Reporting, and Data Integration The gap between evidence generation and clinical adoption has been estimated to exceed a decade based on multiple independent analyses (18, 19), underscoring the need for deliberate, structured mechanisms to accelerate the translation of muscle health research into standardized practice. With this, a layered consensus process is recommended to advance the field: Delphi rounds for discrete elements (e.g., grip strength protocol, ultrasound parameters and positioning) (20–22) and the RAND/UCLA Appropriateness Method for more complex decisions (23). Additionally, the use of standardized reporting templates would serve to capture key technical variables (as shown in Table 1) to improve reproducibility and foster multidisciplinary communication. Consensus on the specific measures appropriate to both clinical and research settings is an important next step for the field.

Step 2: Adopt a Tiered Assessment Approach Begin with a core minimum battery of feasible, high-yield measures spanning each of the three ICF-informed muscle health components: body and tissue composition, muscle performance, and functional task performance. First-tier measures should be broadly accessible, time-efficient, and sufficient to identify individuals at risk or in subclinical stages of muscle health decline to inform whether further assessment is warranted. When clinical context, available resources, or monitoring needs require greater precision, second-tier assessment using advanced imaging, laboratory measures, or instrumented performance testing can be utilized accordingly. This tiered structure is a diagnostic approach that matches assessment complexity to clinical need, supports the proactive identification of at-risk individuals, and may preserve comparability across practice settings. In addition, more resource-intensive modalities may serve a distinct and necessary function in research settings: generating normative data, validating cut-off values, and garnering mechanistic insights.

Step 3: Systematically Address Unresolved Methodological Variation Table 1 highlights persistent sources of heterogeneity that must be addressed for reliable implementation. Key areas of unresolved variation include:

- **Primary Measures**: Even within the same domain, selected variables differ (e.g., appendicular lean mass vs. skeletal muscle index vs. muscle thickness; upper extremity vs. lower extremity strength; gait speed vs. timed sit-to-stand), complicating diagnostic algorithms and the aggregation of results across studies (3, 24).
- **Technical and Protocol Factors**: Substantial differences in device models, operator technique, patient positioning, timing (e.g., time of day, hydration status, post-exercise swelling), and acquisition parameters (e.g., ultrasound gain/TGC settings, MRI sequence type) directly influence measurement reliability and comparability.
- **Cut-off Values and Normative Data**: Cut-offs remain highly variable across guidelines (EWGSOP2, FNIH, AWGS, etc.) and populations, with limited consensus on age-, sex-, or ethnicity-specific thresholds, especially for emerging measures such as ultrasound echogenicity or muscle power (25).
- **Data Interpretation**: Distinguishing among muscle mass/volume and estimates of muscle architecture, fatty infiltration or fibrosis is confounded using some imaging approaches such as DXA (26). Also, determining how to interpret and appropriately weight estimates of muscle mass and muscle composition relative to physical performance and health outcomes remains an unresolved challenge in the field. Regarding physical performance measures, the clinical meaning of a given value often depends on normalization approaches, the presence of comorbidities, and the influence of non-muscle organ systems on functional status (27).

## 5 A call to action

The papers in the Advancing Muscle Health: From Technical and Clinical Research to Practice Research Topic issue represent significant technical and clinical advances concerning the assessment of skeletal muscle. However, the collective works in the journal issue also illustrate how research progress is increasingly constrained by methodological heterogeneity. The growing interest and recently published muscle health frameworks highlights a critical inflection point for the field (4, 5, 28, 29). The next critical phase is formalized consensus development regarding the implementation of muscle health assessments. International organizations, funding bodies, and professional societies will need to support a formal consensus process focused on:

1. Minimum viable muscle health assessment core measures for both clinical and research settings.
2. Standardized outcome measure protocols and training curricula.
3. Clinically meaningful change thresholds and common approaches to normalizing data.
4. Potential integration of medical/biological frameworks for muscle health with the ICF-informed assessment approach in physical rehabilitation settings.
5. Pathways for embedding muscle health metrics into patient-centered care models to build cohesive data sets and support proactive interventions.

Rehabilitation professionals are uniquely qualified to play a leading role in this effort. By adopting a synthesized framework for muscle health that is inclusive of the ICF domains and typology, we can move muscle health assessment from conceptual models to a routine clinical assessment approach, ultimately preserving function, reducing disability, and enhancing quality of life across the lifespan.
